# A small Askin’s tumor presenting with early onset of chest pain

**DOI:** 10.1186/s12957-015-0506-9

**Published:** 2015-03-15

**Authors:** Jin Yong Jeong, Sang Yong Kim, Dae Chul Jeong, Ki Jun Kim

**Affiliations:** Department of Thoracic and Cardiovascular Surgery, Incheon St. Mary’s Hospital, College of Medicine, The Catholic University of Korea, 222 Banpo-Daero, Seocho-Gu, Seoul, 137-701 South Korea; Department of Pediatrics, Incheon St. Mary’s Hospital, College of Medicine, The Catholic University of Korea, 222 Banpo-Daero, Seocho-Gu, Seoul, 137-7 South Korea; Department of Pediatrics, Seoul St. Mary’s Hospital, College of Medicine, The Catholic University of Korea, 222 Banpo-Daero, Seocho-Gu, Seoul, 137-701 South Korea; Diagnostic Radiology, Incheon St. Mary’s Hospital, College of Medicine, The Catholic University of Korea, 222 Banpo-Daero, Seocho-Gu, Seoul, 137-701 South Korea

**Keywords:** Primitive neuroectodermal tumor, Askin’s tumor, Chest wall

## Abstract

Most primitive neuroectodermal tumor of the chest wall destroy the rib, chest wall muscles, diaphragm, and lung or extend into the spinal compartment, resulting in a large-sized tumor and symptoms. In contrast, we recently encountered a rare case of Askin’s tumor presenting with early-onset chest pain despite the small size. After resection of the tumor and adjuvant chemotherapy, the patient remains disease-free over 3 years of follow-up.

## Correspondence

We read with great interest the article by Benbrahim *et al.* [1], which reported two cases of rare primitive neuroectodermal tumor (PNET) developing from the soft tissues of the chest wall (Askin’s tumor). The chest radiograph and computed tomography (CT) of the cases revealed the tumor destroyed the rib cage. The patients received polychemotherapy and one of them surgical resection. We agree that Askin’s tumor is very aggressive and need multimodal treatment, because of the poor prognosis with a high recurrent rate. Recently, we encountered a small Askin’s tumor with early-onset chest pain and present herein.

**Figure 1:**
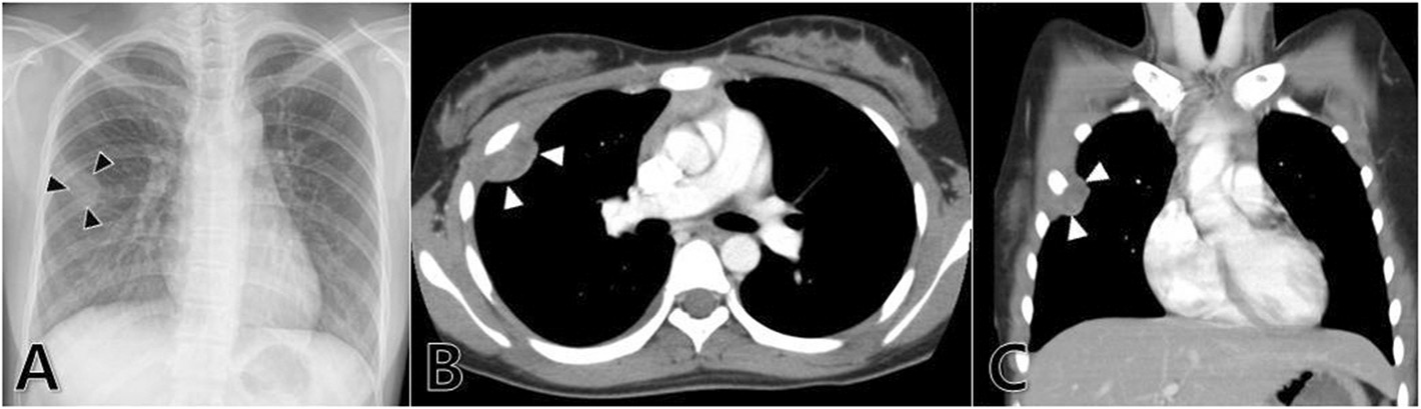
**CT scan revealing a small mass with a broad base.** A small nodular opacity (black arrowheads) in the right chest on chest radiograph **(A)** revealed a mass (2.7 × 2.2 cm in size) on the pleura of the right anterior hemithorax (white arrowheads) with irregular soft tissue density and inner heterogeneous enhancement on CT scan **(B)** and **(C)**.

A 14-year-old girl presented with chest pain. Chest radiograph showed a small nodular opacity in the right lung field, suggesting focal pneumonia or mass, and CT scan revealed a small mass with a broad base on the pleura of the right anterior hemithorax and pleural effusion (Figure 1). We resected the mass for the diagnostic and therapeutic purposes. The pathology and immunohistochemistry of the tumor coincided with PNET which was classified as stage IA(T1aN0M0GX). She received six courses of adjuvant chemotherapy including vincristine, etoposide, doxorubicin, cyclophosphamide, and ifosfamide with mesna. She had been tolerable during chemotherapy, achieved complete response, and has been well without recurrence 3 years thereafter.

Peripheral PNET outside the central nervous system has an insidious onset of symptoms. Askin’s tumor destroys the rib and often extends into the spinal, retroperitoneal, and intrathoracic compartments, resulting in symptoms and a large-sized tumor (>5 cm on average) [2,3]. In contrast, the presenting case is a small-sized Askin’s tumor presenting with early-onset chest pain, which was thought to be caused by the involvement of the parietal pleura. Unfortunately, there is currently no means to early detect an asymptomatic Askin’s tumor. From our case, even a small-sized PNET might cause symptoms. We believe that meticulous examination with CT and/or positron emission tomography and the suspicion of Askin’s tumor prior to surgery is important.

### Consent

Written informed consent was obtained from the patient’s parent for the publication of this report and any accompanying images.

## Abbreviations

CT: computed tomography
PNET: primitive neuroectodermal tumor
